# ^18^F-flortaucipir PET imaging: Biodistribution in the Japanese population and diagnostic accuracy by Japanese readers

**DOI:** 10.1007/s12149-026-02239-4

**Published:** 2026-07-17

**Authors:** Etsuko Imabayashi, Amanda Morris, Leonardo Iaccarino, Anupa K. Arora, Eriko Kamiki, Ming Lu

**Affiliations:** 1 Eli Lilly Japan K.K., Kobe, Japan; 2 Eli Lilly and Company, Indianapolis, IN USA

**Keywords:** Alzheimer’s disease, Neurodegenerative disease, Positron emission tomography, Tauopathies, ^18^F-flortaucipir

## Abstract

**Objective:**

^18^F-flortaucipir (Tauvid™) is an ^18^F-labeled diagnostic positron emission tomography (PET) radiopharmaceutical, which was developed to detect tau pathology in the brain and to estimate the density and/or distribution of aggregated tau neurofibrillary tangles for patients being evaluated for Alzheimer’s disease (AD). The objective of this article is to report findings from two studies. The primary objective of the first *(*dose and biodistribution) study was to investigate the consistency of ^18^F-flortaucipir PET biodistribution between Japanese and non-Japanese individuals. In the second (reader) study, the comparability of visual reads performed by Japanese and US expert readers who followed the same training program was evaluated.

**Methods:**

This article reports findings from two separate studies and datasets. In the dose and biodistribution study, whole-body distribution of ^18^F-flortaucipir was assessed (*N* = 9). Standardized uptake value ratios, normalized to the entire cerebellum, were calculated, and whole-body effective dose was calculated using a 73.7 kg bodyweight model. In the reader study (Study A27; I7E-AV-A27), five Japanese physicians visually interpreted 60 PET scans randomly selected from a previous US reader study (Study FR01; NCT03901092). Scan positivity was determined based on increased activity of ^18^F-flortaucipir in posterolateral temporal, occipital, or parietal/precuneus regions with or without frontal activity. Each scan was visually interpreted by all Japanese readers, who determined whether scans were consistent with an AD or non-AD pattern. Concordance between Japanese and US readers who underwent the same training on the interpretation of ^18^F-flortaucipir PET images was then evaluated (reader study primary endpoint).

**Results:**

The biodistribution of ^18^F-flortaucipir was consistent between Japanese and non-Japanese individuals. When interpreting ^18^F-flortaucipir PET images to distinguish between AD versus non-AD patterns, Japanese and US readers who underwent the same training showed 100% agreement on the majority reads, with a Cohen’s Kappa value of 1.

**Conclusions:**

^18^F-flortaucipir whole-body biodistribution for Japanese individuals was consistent with that of non-Japanese individuals. After completing the same training, Japanese readers showed high concordance with US readers on the interpretation of ^18^F-flortaucipir PET images to distinguish between AD and non-AD patterns. Data and scans were from previous studies registered with ClinicalTrials.gov (registration numbers: NCT04474405; NCT03901092).

**Supplementary Information:**

The online version contains supplementary material available at https://doi.org/10.1007/s12149-026-02239-4.

## Introduction

Alzheimer’s disease (AD) is a neurodegenerative disorder characterized by a progressive decline in cognitive function and ability to perform activities of daily living, possibly leading to death due to complications [1]. Pathologic features of AD include amyloid beta plaques and neurofibrillary tangles (NFTs), which are composed of abnormal accumulations of hyperphosphorylated tau protein, and widespread downstream neurodegeneration [2].

Amyloid positron emission tomography (PET) imaging is a well-established diagnostic tool for the detection of cerebral amyloid pathology in patients with suspected AD [3]. Of note, amyloid accumulation is thought to potentially start well before onset of clinical symptoms, potentially reaching a plateau in the symptomatic phase [4]. In contrast, tau PET imaging has been shown to be more tightly associated with AD symptomatic onset [5], with potential value as a disease staging tool in the symptomatic phase [3].

^18^F-flortaucipir (Tauvid™) is an ^18^F-labeled diagnostic PET radiopharmaceutical developed to detect tau pathology in the brain, particularly in patients being evaluated for AD [6, 7]. After intravenous administration, ^18^F-flortaucipir crosses the blood–brain barrier and selectively binds to aggregated tau proteins, especially the paired helical filaments that form the NFTs, which are characteristic of AD [8]. Once bound to tau aggregates, the radioactive signal from ^18^F-fluorine can be detected using a PET scanner, allowing visualization and estimation of tau density and distribution in the brain.

^18^F-flortaucipir is the first and only PET tracer approved to detect tau pathology associated with AD in adults with cognitive impairment in various geographies, including the United States [9], European Union [10], and United Kingdom [11]. In Japan, ^18^F-flortaucipir has been approved to assist with administration of donanemab, an amyloid-targeting monoclonal antibody [12, 13]. Given ^18^F-flortaucipir uptake indicates tau deposition, which is closely related to impaired cognition and neuronal injury, it has the potential to predict the progression of AD stages and address the unmet medical need in AD care by contributing to clinically meaningful findings [5].

Two pivotal trials evaluated the performance of ^18^F-flortaucipir in estimating the density and distribution of aggregated tau NFTs [14]. Study 1 (A16; NCT02516046; Supplementary Fig. 1) assessed if visual reads of ^18^F-flortaucipir scans corresponded with B3-level (Braak stage V–VI [15]) tau pathology at autopsy. The study included individuals diagnosed with a terminal illness and who were aged > 50 years and had a projected life expectancy of < 6 months. All participants underwent ^18^F-flortaucipir PET imaging, and scans were independently interpreted by five nuclear medicine physicians or radiologists. The ^18^F-flortaucipir PET majority-read interpretations were compared against autopsy results, finding a 92.3% sensitivity and 80.0% specificity in predicting B3 tau pathology [14]. Furthermore, ^18^F-flortaucipir PET scans predicted high AD neuropathology changes (corresponding to advanced amyloid and tau pathology [15]) with 94.7% sensitivity and 80.8% specificity [14]. In Study 2 (Study FR01; NCT03901092; Supplementary Fig. 1), inter-reader agreement as reported using Fleiss' Kappa value was found to be > 0.8 with a new set of readers, evaluating a total of 241 scans, including an autopsy subgroup (*N* = 82) and a cognitively impaired subgroup from an observational study (*N* = 159; Study A05C, NCT02016560 [16]) [14].

In this article, we report two studies designed to: (1) show the consistency of ^18^F-flortaucipir PET whole-body biodistribution between Japanese and non-Japanese individuals (dose and biodistribution study); and (2) assess the comparability of ^18^F-flortaucipir PET visual reads performed by Japanese expert readers or US expert readers who followed the same training program (reader study).

## Materials and methods

### Study design

#### Dose and biodistribution study

To compare the uptake and biodistribution of ^18^F-flortaucipir in Japanese and non-Japanese individuals, data from a phase I open-label exploratory study (NCT04474405) were used. Whole-body PET scans were conducted in healthy volunteers only (Supplementary Methods).

#### Reader study

This reader study (Study A27) was designed to compare reader agreement of the interpretation of ^18^F-flortaucipir PET images between majority visual read results by US readers and those by Japanese readers. Five qualified Japanese physicians were trained in person on the ^18^F-flortaucipir PET scan read methodology; the training consisted of teaching the readers the steps of interpretation, followed by a practice session using a set of demonstration and practice cases and then independently reading 60 scans. Of note, the training program was identical to the training program that had been previously administered to the US readers. The reader study was conducted on a single day (September 2, 2023); database lock was September 21, 2023.

### Ethics

The studies were conducted in accordance with the principles of the Declaration of Helsinki, the International Council for Harmonisation (ICH) Good Clinical Practice (GCP) guidelines, the Code of Federal Regulations, and applicable laws and regulations. Written informed consent was obtained from all healthy volunteers and patients, and from caregivers/guardians if applicable. Participants consented to the use of their data (including scans) for future research purposes.

### Participants

#### Dose and biodistribution study

No new participants were enrolled or imaged for the purposes of this study (Supplementary Methods).

#### Reader study

Five qualified Japanese PET nuclear medicine physicians who were certified by the Japanese Society of Nuclear Medicine and who had experience interpreting brain PET scans but had not received prior training on the ^18^F-flortaucipir read methodology were included in the study.

No new participants were enrolled or imaged for the purposes of this reader study (Study A27). ^18^F-flortaucipir PET scans were collected from a previous reader study (Study FR01, A05C cases; NCT03901092; NCT02016560; Supplementary Fig. 1) [14]. Participants from Study FR01 were male or female, aged ≥ 50 years, with mild cognitive impairment (MCI) or dementia with a suspected neurodegenerative cause, and a Mini Mental State Examination score of 20–27 inclusive.

### Image acquisition

#### Dose and biodistribution study

Images from the phase I open-label exploratory study (NCT04474405) were used. Participants in this study received a single intravenous bolus administration of ~ 370 MBq (10 mCi) of ^18^F-flortaucipir immediately prior to the whole-body scans (Supplementary Methods).

#### Reader study

Scans (*N* = 60) were randomly selected from a previous reader study (Study FR01) [14] using stratified random sampling based on the majority visual read result (non-AD vs. AD). The scans were selected from the A05C cases of Study FR01 because they were comparable to the intended target population in the clinical setting (i.e., participants with MCI and clinically diagnosed AD being evaluated for cognitive decline). Methodology for PET imaging acquisition has been previously published [14]. Briefly, participants underwent PET imaging (4 × 5-minute acquisition frames) beginning ~ 80 min after administration of 370 MBq (± 10%) of intravenous ^18^F-flortaucipir. Frames were motion-corrected and summed from 80 to 100 min after injection.

### Image analysis and assessment

#### Dose and biodistribution study

^18^F-flortaucipir whole-body effective dose in Japanese and non-Japanese individuals was determined. Radiation dose estimates measured in mSv/MBq for the whole body were obtained from Organ Level Internal Dose Assessment/Exponential Modeling (OLINDA/EXM) radiation dosimetry code. Results were calculated using a 73.7 kg bodyweight model.

#### Reader study

All readers were trained in person on the steps of image interpretation using a set of demonstration and practice cases prior to starting their independent reads. After measuring the cerebellar reference region, selecting an appropriate color scale, and scaling the image, regions of the neocortex were visually evaluated for increased tracer uptake.

A scan was deemed positive if there was increased neocortical tracer activity in either/both hemisphere(s) of the posterolateral temporal (PLT), occipital, or parietal/precuneus regions, with or without frontal activity [9]. A scan with a positive AD pattern showed disease progression if increased tracer activity was seen in the parietal (including precuneus) region(s) or frontal region with PLT or occipital regions of either hemisphere.

Each reader made a qualitative assessment of 60 randomly selected ^18^F-flortaucipir PET images, presented in random sequence, to determine AD pattern (τAD+ or τAD++) or non-AD pattern (τAD-) (Table 1). τAD- was defined as no increased neocortical activity, or increased neocortical activity isolated to the mesial temporal, anterolateral temporal, and/or frontal regions. τAD+ was defined by increased accumulation of ¹⁸F-flortaucipir in the PLT or occipital region(s) in either hemisphere. τAD++ was defined by either of the following: increased accumulation of ¹⁸F-flortaucipir in the parietal lobe, including the precuneus region(s), in either hemisphere, whether occurring in isolation or in combination with increased accumulation in the PLT or occipital lobe, or increased accumulation in the frontal lobe accompanied by increased accumulation in the PLT, parietal lobe, or occipital lobe. The reader independently reviewed a PET or PET/computed tomography brain image for each participant and then determined whether the scan was τAD-, τAD+, or τAD++. Readers were blinded to all participant demographic and clinical data and any previous PET scan interpretation (i.e., site investigator, US reader). Readers were also blinded to each other’s assessments.

**Table 1:** Determination of AD pattern

AD pattern (τAD)		Not consistent with AD pattern τAD-
τAD+	τAD++	
In either hemi-sphere, increased accumulation of ^18^F-flortaucipir in the PLT or occipital region(s) Tau positivity with moderate AD tau pattern	In either hemisphere, increased accumulation of ^18^F-flortaucipir in the parietal lobe, including the precuneus region(s) OR Increased accumulation in the frontal lobe in addition to the PLT, the parietal lobe including the precuneus, or the occipital lobe Tau positivity with advanced AD tau pattern	No increased neocortical activity, or increased neocortical activity isolated to the mesial temporal, anterolateral temporal, and/or frontal regions Tau negativity with negative AD pattern

*AD* Alzheimer’s disease, *PLT* posterolateral temporal, *τAD-* neocortical uptake not consistent with AD pattern, *τAD+* moderate AD ^18^F-flortaucipir pattern with increased neocortical activity in the PLT or occipital region(s), *τAD++* advanced AD ^18^F-flortaucipir pattern with increased neocortical activity in the parietal/precuneus region(s), or increased activity in the frontal region(s) accompanied by increases in the PLT, parietal, or occipital region(s)

### Objectives and endpoints

#### Dose and biodistribution study

The objectives were to evaluate the uptake and retention of ^18^F-flortaucipir in the brain, to obtain radiation exposure dose, and to assess whole-body distribution of ^18^F-flortaucipir in Japanese participants compared with non-Japanese participants.

#### Reader study

The primary objective was to evaluate the concordance between Japanese readers and US readers on interpretation of ^18^F-flortaucipir PET images obtained from Study FR01 to distinguish between AD pattern (τAD+/τAD++) and non-AD pattern (τAD-). Primary endpoints were majority visual read results classified as τAD- versus τAD+/τAD++ from Japanese readers and US readers.

The secondary objective was to evaluate the concordance between Japanese readers and US readers on interpretation of the ^18^F-flortaucipir PET images obtained in Study FR01 to distinguish between τAD++ and non-τAD++ (τAD-/τAD+). Secondary endpoints were majority visual read results classified as τAD++ versus non-τAD++ from Japanese readers and US readers.

Exploratory objectives were to evaluate the inter-reader reliability of the ^18^F-flortaucipir scan interpretation across the five Japanese readers for the AD pattern classifications of τAD+/τAD++ versus τAD- and τAD++ versus non-τAD++, and to evaluate the agreement between the majority-read result obtained in Study FR01 and the ^18^F-flortaucipir scan interpretation for each Japanese reader for τAD+/τAD++ versus τAD- and τAD++ versus non-τAD++. Exploratory endpoints were visual read results from Japanese readers classified as τAD+/τAD++ versus τAD- and τAD++ versus non-τAD++, and majority visual read results from US readers classified as τAD+/τAD++ versus τAD- and τAD++ versus non-τAD++.

### Statistical analysis

#### Dose and biodistribution study

Descriptive statistics were applied to summarize the image analysis results.

#### Reader study

Sixty cases were randomly selected using stratified random sampling based on the majority visual read result from Study FR01 (~ 60% tAD+/tAD++ and ~ 40% tAD-). According to the majority rating from five Study FR01 readers, ~ 60% of the 159 Study FR01 (A05C) cases were classified as AD pattern (τAD+/τAD++). Assuming a 60% underlying AD pattern (τAD+/τAD++) rate and a Kappa value expected at 0.9 (almost perfect agreement), a sample of 60 cases would provide > 90% power to detect a difference from Kappa value 0.4 (lower bound of moderate agreement) under a two-sided 5% type I error rate.

The analysis dataset included the ^18^F-flortaucipir visual read results from the five Japanese readers in Study A27 and the corresponding ^18^F-flortaucipir visual read results from the five US readers in Study FR01 for all 60 scans. For analysis, the enrolled set included all 60 cases and the corresponding US readers’ visual read results selected for this reader study. The full analysis set (FAS) included all cases with a valid ^18^F-flortaucipir visual read result from Japanese readers. Data are summarized using descriptive statistics. Simple Cohen’s Kappa statistics, along with two-sided 95% confidence intervals (CIs), were provided to evaluate the consistency between the majority-read results from five Japanese readers and five US readers. The Kappa statistic can be interpreted such that 0 indicates no agreement, 0–0.20 indicates slight agreement, 0.21–0.40 indicates fair agreement, 0.41–0.60 indicates moderate agreement, 0.61–0.80 indicates substantial agreement, and 0.81–1.00 indicates almost perfect agreement [17]. Inter-reader reliability of the ^18^F-flortaucipir scan interpretation (τAD+/τAD++ vs. τAD-) across the five Japanese readers was assessed using Fleiss’ Kappa statistics. The overall percent agreement, Fleiss’ Kappa, and a 95% CI around the Kappa value are provided. Post hoc analyses were performed using the enrolled set to analyze concordance between Japanese readers and US readers on interpretation of ^18^F-flortaucipir images. All analyses were performed using SAS 9.2 or higher (SAS Institute Inc., Cary, NC, USA).

## Results

### Dose and biodistribution study

**Fig. 1:**
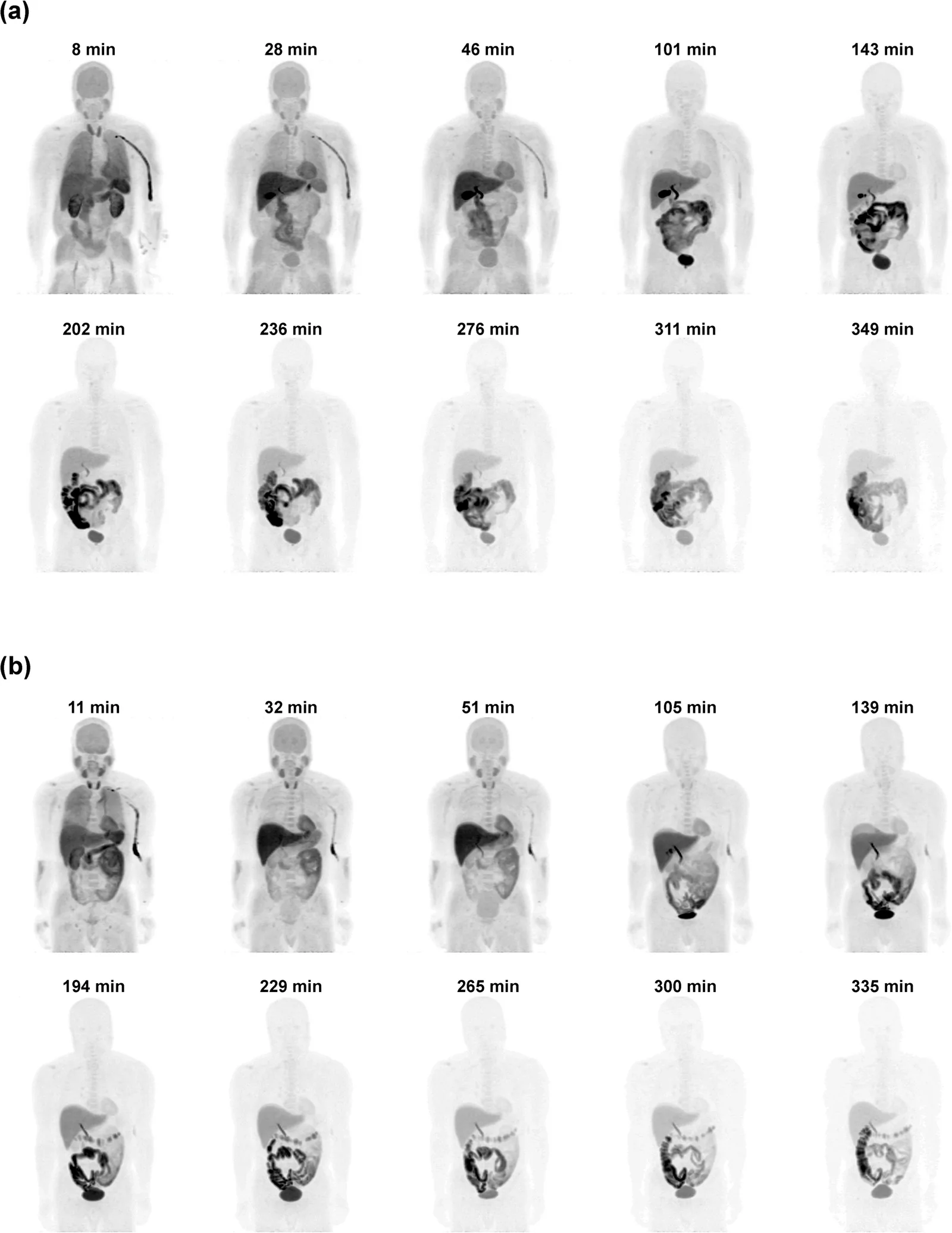
Comparison of Japanese and non-Japanese exposure dose (mean value) estimated by a 73.7 kg bodyweight model (Study A01/A15). Maximum intensity projection images showing representative radioactivity biodistributions after injection of ~10 mCi (370 MBq) of ^18^F-flortaucipir to (**a**) a 58-year-old non-Japanese male and (**b**) a 56-year-old Japanese male

Mean (standard deviation) (mSv/MBq) effective dose equivalent and effective dose, estimated by the 73.7 kg bodyweight model, was 3.07E-02 (1.63E-03) and 2.36E-02 (1.17E-03) in the Japanese population, and 3.21E-02 (2.52E-03) and 2.35E-02 (1.88E-03) in the non-Japanese population (Table 2). The biodistribution of ^18^F-flortaucipir was consistent in the Japanese and non-Japanese populations. ^18^F-flortaucipir was distributed throughout the body, with < 10% of the injected ^18^F radioactivity remaining in the blood at 5 min and < 5% at 10 min post administration. During imaging, ~ 28–34% of circulating ^18^F was parent compound; the rest was metabolites. Clearance occurred primarily by hepatobiliary and renal excretion, with rapid bile excretion observed. An example of whole-body ^18^F-flortaucipir PET images of Japanese and non-Japanese male individuals when infused with 370 MBq of ^18^F-flortaucipir is shown in Fig. 1.

**Table 2:** Estimated corrected exposure dose (mSv/MBq) using the 73.7 kg bodyweight model (Study A01/A15)

	Non-Japanese *N* = 6		Japanese *N* = 3	
	Mean	SD	Mean	SD
Adrenal glands	1.41E-02	9.03E-04	1.44E-02	6.93E-04
Brain	8.23E-03	1.24E-03	8.87E-03	1.24E-03
Breast	7.10E-03	3.13E-04	7.21E-03	2.80E-04
Gallbladder wall	4.12E-02	2.15E-02	3.16E-02	1.19E-02
Lower large intestine wall	3.56E-02	4.84E-03	3.32E-02	3.61E-04
Small intestine	8.68E-02	1.43E-02	7.99E-02	1.04E-03
Stomach lining	1.27E-02	3.20E-04	1.25E-02	3.06E-04
Upper large intestine wall	9.81E-02	1.61E-02	9.02E-02	1.13E-03
Heart wall	2.82E-02	1.72E-03	3.27E-02	4.69E-03
Kidney	4.18E-02	1.22E-02	3.61E-02	5.67E-03
Liver	5.46E-02	8.04E-03	6.25E-02	5.76E-03
Lung	3.40E-02	6.04E-03	3.35E-02	6.37E-03
Muscle	9.00E-03	3.41E-04	8.97E-03	1.87E-04
Ovary	2.10E-02	1.99E-03	2.00E-02	3.21E-04
Pancreas	1.43E-02	6.37E-04	1.45E-02	6.66E-04
Red marrow	1.01E-02	2.69E-04	1.00E-02	2.00E-04
Bone formation cells	1.15E-02	7.23E-04	1.15E-02	1.53E-04
Skin	5.97E-03	3.63E-04	5.98E-03	7.64E-05
Spleen	1.03E-02	5.68E-04	1.01E-02	3.00E-04
Testis	6.93E-03	5.38E-04	6.97E-03	1.98E-04
Thymus	8.52E-03	4.07E-04	8.69E-03	3.76E-04
Thyroid gland	6.71E-03	5.47E-04	6.72E-03	1.58E-04
Urinary bladder wall	3.57E-02	9.94E-03	4.15E-02	1.37E-02
Womb	1.84E-02	1.44E-03	1.79E-02	8.00E-04
Systemic	1.19E-02	2.07E-04	1.19E-02	3.61E-04
Effective dose equivalent	3.21E-02	2.52E-03	3.07E-02	1.63E-03
Effective dose	2.35E-02	1.88E-03	2.36E-02	1.17E-03

*mSv/MBq* millisieverts per megabecquerel, *SD* standard deviation

### Reader study

#### Demographic and baseline clinical characteristics

The enrolled set consisted of 60 cases, and the full analysis set consisted of 56 cases; four cases were uninterpretable by Japanese readers due to noise, low count density, or attenuation correction error and were therefore excluded from the FAS. Baseline demographics for the enrolled set are presented in Table 3. Mean age was 71.6 years, 98.3% of cases were of White race, 43.3% were female, 75.0% had ≥ 13 years of education, and 55.0% had a clinical diagnosis of MCI.

**Table 3:** Demographics and baseline characteristics of cases selected for Study A27 (FAS)

	Majority interpretation results from US readers		
	τAD+/τAD++ (*N* = 35)	τAD- (*N* = 25)	Overall (*N* = 60)
Female sex, *n* (%)	18 (51.4)	8 (32.0)	26 (43.3)
Mean age, years (SD)	73.5 (7.4)	68.9 (11.2)	71.6 (9.3)
Race, *n* (%)			
Black or African American	0	1 (4.0)	1 (1.7)
White	35 (100)	24 (96.0)	59 (98.3)
Ethnicity, Hispanic/Latino, *n* (%)	2 (5.7)	1 (4.0)	3 (5.0)
Education, ≥ 13 years, *n* (%)	27 (77.1)	18 (72.0)	45 (75.0)
Clinical diagnosis group, *n* (%)			
MCI	15 (42.9)	18 (72.0)	33 (55.0)
AD	20 (57.1)	7 (28.0)	27 (45.0)

*AD* Alzheimer’s disease, *FAS* full analysis set, *MCI* mild cognitive impairment, *PLT* posterolateral temporal, *SD* standard deviation, *τAD-* neocortical uptake not consistent with AD pattern, *τAD+* moderate AD ^18^F-flortaucipir pattern with increased neocortical activity in the PLT or occipital region(s), *τAD++* advanced AD ^18^F-flortaucipir pattern with increased neocortical activity in the parietal/precuneus region(s), or increased activity in the frontal region(s) accompanied by increases in the PLT, parietal, or occipital region(s)

#### Primary endpoint

The majority reads from Japanese and US readers showed 100% agreement with a Cohen’s Kappa value of 1 when interpreting ^18^F-flortaucipir PET images to distinguish between AD pattern (τAD+/τAD++) versus non-AD pattern (τAD-) (Table 4). The majority imaging visual interpretation results of all Japanese and US readers for AD pattern (τAD+/τAD++) and non-AD pattern (τAD-) are shown in Table 4.

**Table 4:** Concordance between Japanese readers and US readers on interpretation of ^18^F-flortaucipir images (τAD+/τAD++ vs. τAD-) (FAS)

Majority read: Japanese readers	Majority read: US readers		
	τAD+/τAD++	τAD-	Total
τAD+/τAD++	35	0	35
τAD-	0	21	21
Total	35	21	56
**Overall agreement**			
Cohen’s Kappa (95% CI)		1 (1.0–1.0)	
*p*-value^a^		< 0.001	
Overall percent agreement (95% CI^b^)		100 (93.6–100.0)	

^a^The *p*-value corresponds to the test with null hypothesis: Kappa = 0

^b^The 95% CI is based on the Wilson score method

*AD* Alzheimer’s disease, *CI* confidence interval, *FAS* full analysis set, *PLT* posterolateral temporal, *τAD-* neocortical uptake not consistent with AD pattern, *τAD+* moderate AD ^18^F-flortaucipir pattern with increased neocortical activity in the PLT or occipital region(s), *τAD++* advanced AD ^18^F-flortaucipir pattern with increased neocortical activity in the parietal/precuneus region(s), or increased activity in the frontal region(s) accompanied by increases in the PLT, parietal, or occipital region(s)

#### Secondary endpoint

The majority reads from Japanese and US readers showed 100% agreement and a Cohen’s Kappa value of 1 when interpreting ^18^F-flortaucipir images to distinguish between τAD++ and non-τAD++ (τAD+/τAD-) (Table 5).

**Table 5:** Concordance between Japanese readers and US readers on interpretation of ^18^F-flortaucipir images (τAD++ vs. non-τAD++) (FAS)

Majority read: Japanese readers	Majority read: US readers		
	τAD++	τAD+/τAD-	Total
τAD++	31	0	31
τAD+/τAD-	0	25	25
Total	31	25	56
**Overall agreement**			
Cohen’s Kappa (95% CI)		1 (1.0–1.0)	
*p*-value^a^		< 0.001	
Overall percent agreement (95% CI^b^)		100 (93.6–100.0)	

^a^The *p*-value corresponds to the test with null hypothesis: Kappa = 0

^b^The 95% CI is based on the Wilson score method

*AD* Alzheimer’s disease, *CI* confidence interval, *FAS* full analysis set, *PLT* posterolateral temporal, *τAD-* neocortical uptake not consistent with AD pattern, *τAD+* moderate AD ^18^F-flortaucipir pattern with increased neocortical activity in the PLT or occipital region(s), *τAD++* advanced AD ^18^F-flortaucipir pattern with increased neocortical activity in the parietal/precuneus region(s), or increased activity in the frontal region(s) accompanied by increases in the PLT, parietal, or occipital region(s)

#### Exploratory endpoints

When distinguishing between AD pattern (τAD+/τAD++) versus non-AD pattern (τAD-) images, Japanese readers displayed an overall percent agreement of 95.7% and a Fleiss’ Kappa value of 0.91, indicating almost perfect consistency between readers and image interpretations (Table 6). Inter-reader reliability across the five Japanese readers is shown in Supplementary Table 1. When distinguishing between τAD++ and non-τAD++ (τAD-/τAD+) images, Japanese readers displayed an overall percent agreement of 95.4% and a Fleiss’ Kappa value of 0.91, indicating almost perfect consistency between readers and image interpretations (Table 6). Inter-reader reliability across the five Japanese readers is shown in Supplementary Table 2.

**Table 6:** Inter-reader reliability across Japanese readers (FAS)

Overall agreement between Japanese readers	
τAD+/τAD++ vs. τAD-	
Fleiss’ Kappa (95% CI)	0.91 (0.83–0.99)
* p*-value^a^	< 0.001
Overall percent agreement (95% CI)	95.7 (93.7–97.1)
τAD++ vs. non-τAD++	
Fleiss’ Kappa (95% CI)	0.91 (0.83–0.99)
* p*-value^a^	< 0.001
Overall agreement (95% CI)	95.4 (93.3–96.8)

^a^The *p*-value corresponds to the test with null hypothesis: Kappa = 0

*AD* Alzheimer’s disease, *CI* confidence interval, *FAS* full analysis set, *PLT* posterolateral temporal, *τAD-* neocortical uptake not consistent with AD pattern, *τAD+* moderate AD ^18^F-flortaucipir pattern with increased neocortical activity in the PLT or occipital region(s), *τAD++* advanced AD ^18^F-flortaucipir pattern with increased neocortical activity in the parietal/precuneus region(s), or increased activity in the frontal region(s) accompanied by increases in the PLT, parietal, or occipital region(s)

Each Japanese reader showed high concordance with the majority-read results obtained from Study FR01 when interpreting ^18^F-flortaucipir images to distinguish between τAD+/τAD++ versus τAD- (Table 7) and τAD + + versus non-τAD++ (Table 8).

**Table 7:** Concordance between Japanese readers and Study FR01 majority-read results (τAD+/τAD++ vs. τAD-) (FAS)

Japan reader	Agreement between Japanese and majority read from Study FR01		
	% Agreement with FR01 reads (95% CI^a^)	Cohen’s Kappa (95% CI)	*p*-value^b^
1	96.4 (87.9–99.0)	0.92 (0.82–1.0)	< 0.001
2	98.2 (90.6–99.7)	0.96 (0.89–1.0)	< 0.001
3	94.6 (85.4–98.2)	0.89 (0.77–1.0)	< 0.001
4	100.0 (93.6–100.0)	1.0 (1.0–1.0)	< 0.001
5	98.2 (90.6–99.7)	0.96 (0.89–1.0)	< 0.001

^a^The 95% CI is based on the Wilson score method

^b^The *p*-value corresponds to the test with null hypothesis: Kappa = 0

*AD* Alzheimer’s disease, *CI* confidence interval, *FAS* full analysis set, *PLT* posterolateral temporal, *τAD-* neocortical uptake not consistent with AD pattern, *τAD+* moderate AD ^18^F-flortaucipir pattern with increased neocortical activity in the PLT or occipital region(s), *τAD++* advanced AD ^18^F-flortaucipir pattern with increased neocortical activity in the parietal/precuneus region(s), or increased activity in the frontal region(s) accompanied by increases in the PLT, parietal, or occipital region(s)

**Table 8:** Concordance between Japanese readers and Study FR01 majority-read results (τAD++ vs. non-τAD++) (FAS)

Japan reader	Agreement between Japanese and majority read from Study FR01		
	% Agreement with FR01 reads (95% CI^a^)	Cohen’s Kappa (95% CI)	*p*-value^b^
1	94.6 (85.4–98.2)	0.89 (0.77–1.0)	< 0.001
2	94.6 (85.4–98.2)	0.89 (0.77–1.0)	< 0.001
3	98.2 (90.6–99.7)	0.96 (0.89–1.0)	< 0.001
4	98.2 (90.6–99.7)	0.96 (0.89–1.0)	< 0.001
5	100.0 (93.6–100.0)	1.0 (1.0–1.0)	< 0.001

^a^The 95% CI is based on the Wilson score method

^b^The *p*-value corresponds to the test with null hypothesis: Kappa = 0

*AD* Alzheimer’s disease, *CI* confidence interval, *FAS* full analysis set, *PLT* posterolateral temporal, *τAD++* advanced AD ^18^F-flortaucipir pattern with increased neocortical activity in the parietal/precuneus region(s), or increased activity in the frontal region(s) accompanied by increases in the PLT, parietal, or occipital region(s)

#### Post hoc analyses

Japanese and US readers showed 100% agreement and a Cohen’s Kappa of 1 when interpreting ^18^F-flortaucipir images to distinguish between AD pattern (τAD+/τAD++) and non-AD pattern (τAD-) (Supplementary Table 3 [enrolled set]), and between τAD++ and non-τAD++ (τAD-/τAD+) (Supplementary Table 4 [enrolled set])

## Discussion

The studies reported in this article evaluated the radiation exposure dose of ^18^F-flortaucipir in Japanese participants and provide evidence that Japanese readers’ performance in interpreting ^18^F-flortaucipir PET images was comparable to that observed for US expert readers on the same dataset after completing the same training. In the dose and biodistribution study, imaging data obtained from Japanese individuals were similar to those of non-Japanese individuals pertaining to dose of radiation exposure and biodistribution. In the reader study, majority-read results from Japanese readers showed high concordance with US expert readers on the interpretation of ^18^F-flortaucipir PET images to distinguish between AD and non-AD patterns, and between τAD++ and non-τAD++ (τAD+/τAD-). Japanese readers showed high inter-reader reliability on interpretation of ^18^F-flortaucipir PET images, and each Japanese reader also showed high concordance with the majority-read results obtained from Study FR01. The results of the current study demonstrate that Japanese readers have high concordance with US readers on the interpretation of ^18^F-flortaucipir PET images when given the same training, supporting the safe use of ^18^F-flortaucipir in Japan.

Alzheimer’s disease is characterized by amyloid beta plaques and aggregated tau NFTs, which can appear years before symptoms. These pathologic markers, previously only detectable post mortem, can now be identified in vivo using biomarkers, thereby allowing for a definitive diagnosis [7]. ^18^F-flortaucipir has been extensively validated in various research and clinical settings, highlighting its diagnostic and prognostic value in typical and atypical AD, MCI, and pre-symptomatic stages, with limited evidence for other neurodegenerative disorders [7]. Despite varied interpretation methods, the literature supports ^18^F-flortaucipir’s high value in AD diagnosis and prognosis [7]. Recently, expert recommendations for the implementation of amyloid and tau PET in clinical practice for guiding the diagnosis and management of patients who have, or who are at risk for, cognitive decline have been reported [18], further supporting the clinical application of ^18^F-flortaucipir. Although ^18^F-flortaucipir has quantitatively been shown to be potentially sensitive enough to identify Braak stage IV in the inferior temporal region [19], the visual read method has been validated to detect advanced tau pathology (B3-level, Braak stage V or VI [15]).

A key strength of the dose and biodistribution study is the diverse set of images sourced from multiple studies, offering a realistic reflection of those encountered in daily clinical practice. The inclusion of Japanese participants in the dose and biodistribution study shows valid evidence of its safe use in the Japanese population. However, we acknowledge that the study included participants of a relatively young age range (> 50 years), and only three out of a total of nine participants were Japanese, which limits the extent to which the dose and biodistribution findings can be generalized to the Japanese population. Nevertheless, exposure dose results from Choi et al. [20], whose study comprised six healthy Korean participants, yielded similar findings. Taken together, the consistent results observed in both Japanese and Korean participants support the safe use of ^18^F-flortacuipir in the Japanese population.

In vitro, titrated flortaucipir has been reported to bind with low nanomolar affinity to monoamine oxidase (MAO)-A and MAO-B, which could contribute to off-target binding [9]. While in vitro studies have reported higher binding affinity of ¹⁸F-flortaucipir for MAO-A than MAO-B, the in vivo relevance of MAO-A–related binding remains limited. Enzyme inhibition studies have demonstrated that MAO-related binding is not expected to significantly affect cortical ¹⁸F-flortaucipir signals [21], and modeling work corroborates that MAO-related binding is unlikely to meaningfully impact typical clinical interpretation [9]. Importantly, a recent voxel-to-voxel histology-to-PET study [22] provides direct spatial evidence that, in AD, phosphorylated tau burden is the dominant contributor to the ¹⁸F-flortaucipir signal, with an ~ 1.5-fold greater regression coefficient than ferric iron, and with MAO-B showing an inverse association in cortical regions. The same study demonstrated that MAO-B signal is predominantly concentrated in white matter, thereby limiting its influence on neocortical ¹⁸F-flortaucipir interpretation. These findings collectively support the conclusion that, while off-target binding to iron and MAOs exists, it does not substantially compromise the ability of ¹⁸F-flortaucipir to reflect AD tau pathology in neocortical regions relevant to clinical visual read interpretation.

The near-perfect consistency of read results by Japanese readers underscores the technical robustness of ^18^F-flortaucipir for clinical use. Like in any reader study, however, this study was carried out in a controlled environment, which may not fully capture the complexity and pressures of actual clinical practice; nevertheless, similar high concordance for the ^18^F-flortaucipir visual read method was also reported in Europe [23]. Together, these results support the reliability of using ^18^F-flortaucipir as a PET tracer regardless of geography and associated clinical environment. As such, despite limitations, it is reasonable to infer that ^18^F-flortaucipir PET tracer visual read can be performed with precision by Japanese readers given the relevant training.

## Conclusion

The biodistribution of ^18^F-flortaucipir throughout the body was equivalent in Japanese and non-Japanese populations. Japanese readers showed high concordance among Japanese readers, and with US readers, on the interpretation of ^18^F-flortaucipir PET images to distinguish between AD and non-AD patterns, thereby demonstrating that given the same training, Japanese physicians were able to read ^18^F-flortaucipir PET images with the same high precision as US physicians.

## Supplementary Information

Below is the link to the electronic supplementary material.

Supplementary Material 1

## Data Availability

Data sharing is not applicable to this article as no datasets were generated or analyzed during the current study.
