# Supplementary material for: 18F-flortaucipir PET imaging: Biodistribution in the Japanese population and diagnostic accuracy by Japanese readers
Source: Ann Nucl Med. 2026 Jul 17;40(10):1219–28. doi: 10.1007/s12149-026-02239-4 (PMC13619764; doi:10.1007/s12149-026-02239-4)
Supplement: Supplementary file 1 [file 12149_2026_2239_MOESM1_ESM.pdf]

SUPPLEMENTARY MATERIAL

^{18}F-flortaucipir PET imaging: biodistribution in the Japanese population and diagnostic accuracy by Japanese readers

Annals of Nuclear Medicine

Etsuko Imabayashi[1], Amanda Morris[2], Leonardo Iaccarino[2], Anupa K. Arora[2], Eriko Kamiki[1], Ming Lu[2]

[1] Eli Lilly Japan K.K., Kobe, Japan

[2] Eli Lilly and Company, Indianapolis, IN, United States

Correspondence:

Etsuko Imabayashi

Email: etsuko.imabayashi@lilly.com

LIST OF TABLES AND FIGURES

Supplementary Methods

Supplementary Table 1 Inter-reader reliability across Japanese readers (τAD+/τAD++ *vs.* τAD-) (FAS)

Supplementary Table 2 Inter-reader reliability across Japanese readers (τAD++ *vs.* non-τAD++) (FAS)

Supplementary Table 3 Concordance between Japanese readers and US readers on interpretation of ^{18}F-flortaucipir images (τAD+/τAD++ *vs.* τAD-) (enrolled set)

Supplementary Table 4 Concordance between Japanese readers and US readers on interpretation of ^{18}F-flortaucipir images (τAD++ *vs.* non-τAD++) (enrolled set)

Supplementary Fig. 1 Study 1 (A16) and Study 2 (FR01 and A05C cases)

Supplementary Methods

Study design

Dose and biodistribution study

Positron emission tomography (PET) whole-body biodistribution cohort

Nine healthy volunteers were to participate in the biodistribution cohort. Three of these participants were to be first-generation Japanese. Healthy volunteers who qualified had a catheter placed for intravenous (IV) administration of ^{18}F-flortaucipir injection. When the catheter was in place, the participant lay on the scanner bed, and a whole-body transmission scan (computed tomography [CT] scan if using PET/CT) was performed. When the scanner bed was in the PET position, the participant was injected with a single IV bolus of approximately 370 MBq (10 mCi) (±10%) of ^{18}F-flortaucipir followed by a saline flush. Immediately following the injection of ^{18}F-flortaucipir, approximately 10 emission scans from the vertex of the head to the thighs were repeated over a period of about 6 hours to assess the distribution of radioactivity in the body. The participant was asked to void their bladder during scheduled breaks in the imaging session. Food intake during the imaging visit was recorded. Adverse events were continuously monitored. Based on urine excretion rate calculated from obtained urine samples, radiation dosimetry estimates from 18F-AV-1451-A01 study were refined.

Selection of participants: inclusion criteria

1. Males or females aged ≥50 years;
2. Able to tolerate a series of whole-body PET scans to occur over a 6-hour period. The Investigator carefully assessed each participant and used sound medical judgment to determine whether the participant could tolerate the PET scan procedure; and
3. Provided informed consent for study procedures.

Three of the healthy volunteers were to be Japanese, and six of the healthy volunteers were to be non-Japanese.

<u>Selection of participants: exclusion criteria</u>

1. Had current clinically significant psychiatric disease;

2. Had evidence of structural abnormalities such as major stroke or mass on magnetic resonance imaging (MRI) that made a diagnosis of impairment due to AD unlikely or was likely to interfere with interpretation of a PET scan on MRI;

3. Were claustrophobic or otherwise unable to tolerate the imaging procedure;

4. Had current clinically significant cardiovascular disease or clinically significant abnormalities on screening electrocardiogram (including but not limited to QTc>450 msec);

5. Had a current clinically significant infectious disease, endocrine or metabolic disease, pulmonary, renal, or hepatic impairment, or cancer that the Investigator believes would affect study participation or scan results;

6. Had a history of alcohol abuse or substance abuse or dependence;

7. Were females of childbearing potential who were not surgically sterile, not refraining from sexual activity, or not using reliable methods of contraception. Females of childbearing potential were not pregnant (negative serum beta-human chorionic gonadotropin [β-hCG] at the time of screening and negative urine β-hCG within 24 hours prior to injection) or breastfeeding at screening. Females were to agree to avoid becoming pregnant, and both females and males were to agree to refrain from sexual activity or to use reliable contraceptive methods for 90 days following administration of ^{18}F-flortaucipir;

8. Required medications with a narrow therapeutic window (e.g., warfarin);

9. Had a non–study-related radiopharmaceutical imaging or treatment procedure within 7 days prior to the ^{18}F-flortaucipir or florbetapir (^{18}F) study imaging session;

10. Were receiving any investigational medications, or had participated in a trial with investigational medications within the last 30 days;

11. Treatment with psychotropic medication was not forbidden, but Investigators were to discuss with the sponsor and carefully consider whether participants requiring psychotropic medications would be able to complete the imaging session;

12. In the opinion of the Investigator, are otherwise unsuitable for a study of this type.

Supplementary Table 1 Inter-reader reliability across Japanese readers (τAD+/τAD++ *vs.* τAD-) (FAS)

	Agreement between two Japanese readers	
Reader	% Agreement between two readers (95% CI)	Cohen's Kappa (95% CI)
	**Reader 1**	
2	94.6 (85.4–98.2)	0.89 (0.76–1.0)
3	91.1 (80.7–96.1)	0.81 (0.66–0.97)
4	96.4 (87.9–99.0)	0.92 (0.82–1.0)
5	94.6 (85.4–98.2)	0.89 (0.76–1.0)
	**Reader 2**	
3	96.4 (87.9–99.0)	0.93 (0.83–1.0)
4	98.2 (90.6–99.7)	0.96 (0.89–1.0)
5	96.4 (87.9–99.0)	0.93 (0.82–1.0)
	**Reader 3**	
4	94.6 (85.4–98.2)	0.89 (0.77–1.0)
5	96.4 (87.9–99.0)	0.93 (0.83–1.0)
	**Reader 4**	
5	98.2 (90.6–99.7)	0.96 (0.89–1.0)

AD Alzheimer's disease, *CI* confidence interval, *FAS* full analysis set, *PLT* posterolateral temporal, *τAD-* neocortical uptake not consistent with AD pattern, *τAD+* moderate AD ^{18}F-flortaucipir pattern with increased neocortical activity in the PLT or occipital region(s), *τAD++* advanced AD ^{18}F-flortaucipir pattern with increased neocortical activity in the parietal/precuneus region(s), or increased activity in the frontal region(s) accompanied by increases in the PLT, parietal, or occipital region(s)

Supplementary Table 2 Inter-reader reliability across Japanese readers (τAD++ *vs.* non-τAD++) (FAS)

	Agreement between two Japanese readers	
Reader	% Agreement between two readers (95% CI)	Cohen's Kappa (95% CI)
	**Reader 1**	
2	92.9 (83.0–97.2)	0.85 (0.72–0.99)
3	92.9 (83.0–97.2)	0.85 (0.72–0.99)
4	96.4 (87.9–99.0)	0.93 (0.83–1.0)
5	94.6 (85.4–98.2)	0.89 (0.77–1.0)
	**Reader 2**	
3	96.4 (87.9–99.0)	0.93 (0.83–1.0)
4	92.9 (83.0–97.2)	0.86 (0.72–0.99)
5	94.6 (85.4–98.2)	0.89 (0.77–1.0)
	**Reader 3**	
4	96.4 (87.9–99.0)	0.93 (0.83–1.0)
5	98.2 (90.6–99.7)	0.96 (0.89–1.0)
	**Reader 4**	
5	98.2 (90.6–99.7)	0.96 (0.89–1.0)

AD Alzheimer's disease, *CI* confidence interval, *FAS* full analysis set, *PLT* posterolateral temporal, *τAD++* advanced AD ^{18}F-flortaucipir pattern with increased neocortical activity in the parietal/precuneus region(s), or increased activity in the frontal region(s) accompanied by increases in the PLT, parietal, or occipital region(s)

Supplementary Table 3 Concordance between Japanese readers and US readers on interpretation of ^{18}F-flortaucipir images (τAD+/τAD++ *vs.* τAD-) (enrolled set)

Majority read: Japanese readers	Majority read: US readers		
	τAD+/τAD++	τAD-	Total
τAD+/τAD++	35	0	35
τAD-	0	25	25
Total	35	25	60
**Overall agreement**			
Cohen's Kappa (95% CI)		1 (1.0–1.0)	
p-value[a]		<0.001	
Overall percent agreement (95% CI[b])		100 (94.0–100.0)	

[a]The *p*-value corresponds to the test with null hypothesis: Kappa = 0
[b]The 95% CI is based on the Wilson score method
AD Alzheimer's disease, *CI* confidence interval, *PLT* posterolateral temporal, *τAD-* neocortical uptake not consistent with AD pattern, *τAD+* moderate AD ^{18}F-flortaucipir pattern with increased neocortical activity in the PLT or occipital region(s), *τAD++* advanced AD ^{18}F-flortaucipir pattern with increased neocortical activity in the parietal/precuneus region(s), or increased activity in the frontal region(s) accompanied by increases in the PLT, parietal, or occipital region(s)

Supplementary Table 4 Concordance between Japanese readers and US readers on interpretation of ^{18}F-flortaucipir images (τAD++ *vs.* non-τAD++) (enrolled set)

Majority read: Japanese readers	Majority read: US readers		
	τAD++	τAD+/τAD-	Total
τAD++	31	0	31
τAD+/τAD-	0	29	29
Total	31	29	60
**Overall agreement**			
Cohen's Kappa (95% CI)		1 (1.0–1.0)	
p-value[a]		<0.001	
Overall percent agreement (95% CI[b])		100 (94.0–100.0)	

[a]The *p*-value corresponds to the test with null hypothesis: Kappa = 0
[b]The 95% CI is based on the Wilson score method
AD Alzheimer's disease, *CI* confidence interval, *PLT* posterolateral temporal, *τAD-* neocortical uptake not consistent with AD pattern, *τAD+* moderate AD ^{18}F-flortaucipir pattern with increased neocortical activity in the PLT or occipital region(s), *τAD++* advanced AD ^{18}F-flortaucipir pattern with increased neocortical activity in the parietal/precuneus region(s), or increased activity in the frontal region(s) accompanied by increases in the PLT, parietal, or occipital region(s)

Supplementary Fig. 1 Study 1 (A16) and Study 2 (FR01 and A05C cases)

The performance of flortaucipir imaging to estimate the density and distribution of aggregated tau NFTs was evaluated in two clinical studies: Study 1 and Study 2 [1]. In each study, flortaucipir was interpreted by five independent readers blinded to clinical information; readers interpreted flortaucipir imaging as positive or negative [1].

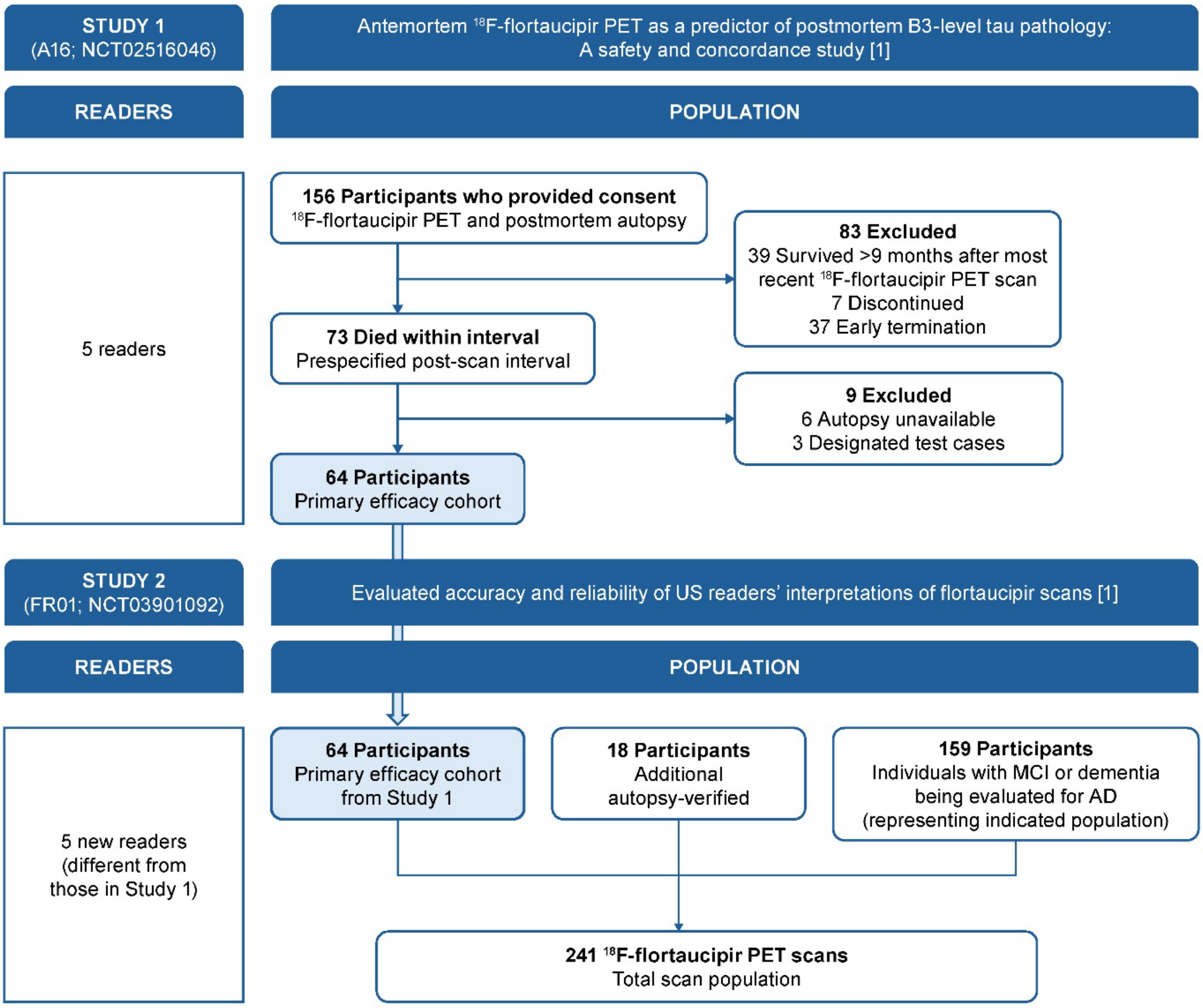

AD Alzheimer's disease, *MCI* mild cognitive impairment, *NFT* neurofibrillary tangles; *PET* positron emission tomography

1. Fleisher AS, Pontecorvo MJ, Devous MD, Sr., Lu M, Arora AK, Truocchio SP, et al. Positron emission tomography imaging with [18F]flortaucipir and postmortem assessment of Alzheimer disease neuropathologic changes. JAMA Neurol 2020;77(7):829–39.
